# Valorization of Wastewater from Table Olives: NMR Identification of Antioxidant Phenolic Fraction and Microwave Single-Phase Reaction of Sugary Fraction

**DOI:** 10.3390/antiox10111652

**Published:** 2021-10-21

**Authors:** Alberto J. Huertas-Alonso, Mohsen Gavahian, Diego J. González-Serrano, Milad Hadidi, Manuel Salgado-Ramos, M. Prado Sánchez-Verdú, Mario J. Simirgiotis, Francisco J. Barba, Daniel Franco, José M. Lorenzo, Andrés Moreno

**Affiliations:** 1 Department of Organic Chemistry, Faculty of Chemical Sciences and Technologies (San Alberto Magno Building), University of Castilla-La Mancha, Av. Camilo José Cela, 10, 13071 Ciudad Real, Spain; alberto.halonso@uclm.es (A.J.H.-A.); DiegoJesus.Gonzalez@uclm.es (D.J.G.-S.); Milad.Hadidi@uclm.es (M.H.); manuel.salgado@uclm.es (M.S.-R.); mariaprado.sanchez@uclm.es (M.P.S.-V.); 2 Department of Food Science, National Pingtung University of Science and Technology, 1, Shuefu Road, Neipu, Pingtung 91201, Taiwan; mohsengavahian@yahoo.com; 3 Institute of Pharmacy, Faculty of Sciences, Campus Isla Teja, Universidad Austral de Chile, Valdivia 5090000, Chile; mario.simirgiotis@uach.cl; 4 Nutrition and Food Science Area, Preventive Medicine and Public Health, Food Science, Toxicology and Forensic Medicine Department, Faculty of Pharmacy, Universitat de València, Av. Vicent Andrés Estellés, s/n, Burjassot, 46100 València, Spain; francisco.barba@uv.es; 5 Centro Tecnológico de la Carne de Galicia, Av. Galicia No. 4, Parque Tecnológico de Galicia, San Cibrao das Viñas, 32900 Ourense, Spain; jmlorenzo@ceteca.net; 6 Área de Tecnología de los Alimentos, Facultad de Ciencias de Ourense, Universidad de Vigo, 32004 Ourense, Spain

**Keywords:** waste valorization, table olive wastewater, NMR, phenolic compounds, levulinic acid, HMF, resource efficiency

## Abstract

The table olive industry is producing a huge amount of wastewater, which is a post-processing cost and an environmental concern. The present study aims to valorize this processing by-product to obtain a value-added product, thereby enhancing resource efficiency and contributing to achieving sustainable development goals (SDGs). In this sense, a chemical reaction-based platform was developed to obtain valuable components, such as levulinic acid (LA) and 5-hydromethylfurfural (HMF). The products were then analyzed using NMR identification of the antioxidant phenolic fraction and microwave single-phase reaction of the sugary fraction. According to the results, the highest concentration of phenolic compounds does not correspond to the sample directly obtained from NaOH treatment (S1), indicating that water washing steps (S2–S5) are fundamental to recover phenolic substances. Moreover, glucose was presented in the sugary fraction that can be transformed into levulinic acid by a single-phase reaction under microwave irradiation. The information provided in this manuscript suggests that the wastewater from the olive processing industry can be valorized to obtain valuable products.

## 1. Introduction

The table olive industry annually produces around 3 million tons of olives, with the European Union contributing around 25%. This high level of production results in a large amount of wastewater, which then poses a threat to the environment due to its high salinity, conductivity, and organic matter levels, as well as its phenolic contents. The problem of processing this wastewater is mainly focused on the Mediterranean area, where most table olives are produced, and to a lesser extent in the USA and South America. The table olive industry has a significant economic impact on Mediterranean countries, with Spain and Greece its main contributors. Spain, for example, generates around 540,000 tons of wastewater from olive processing [1]. It is also worth noting the important production of other Mediterranean countries such as Egypt, Turkey, Algeria, and Morocco, which together comprise about 50% of global production [2].

The fruit of the olive tree is a popular food around the world with several health benefits [3,4,5]. This fruit cannot be consumed raw due to its strong bitter taste. This flavor is usually attributed to the presence of oleuropein, which is the most abundant phenolic compound in raw olives. Along with oleuropein, other seco-iridoids glycosides present in olives, although in smaller quantities, are ligustrazine and demethyloleuropein [6]. In Spanish- and Californian-style processing methods, washing with sodium hydroxide (NaOH) followed with rinsing with water is used to hydrolyze oleuropein and eliminate the bitterness to then make olives edible fruit, releasing interesting phenolic compounds in the process. Alternatively, the Greek-style method has recently gained some popularity as it avoids using chemicals, such as NaOH, and instead uses several months of water (brine) treatment [7].

Recent research has highlighted the importance of valorization of waste from the agri-food industry for the production of value-added products, as well as for meeting the environmental regulations that contribute to achieving sustainable development goals (SDGs) through enhancing resource efficiency [8,9,10,11,12,13]. This includes the use of different techniques to obtain the valuable components of waste that are produced during agri-food processes [5,14,15]. It should be noted that, due to the high content of phenolic compounds in olives, wastewater from olive processing can be a source of these valuable compounds.

Phenolic compounds are bioactive molecules with high antioxidant activity and health benefits, which generate great interest in the food, pharmaceutical and cosmetic industries [16]. These compounds can act as radical scavengers and are formed in the different metabolic processes of cell walls. Free radicals are highly reactive and unstable chemical species which, in excess, can induce lipid and protein oxidation; alter cell membrane integrity; and damage DNA to cause serious pathologies, such as cancer, Parkinson’s disease, diabetes, and Alzheimer’s disease [17]. Therefore, an external source of antioxidants can help in preventing cellular oxidative stress and its consequences [17]. Consequently, the recovery of phenolic components from olive processing wastewater can provide industrial benefits. Most industries employ systems to remove suspended solids and to discolor table olive processing wastewater, such as active carbon absorption and ultrafiltration through membranes of a certain pore size. These systems reduce the pollutant nature of wastewater but, at the same time, diminish the concentration of valuable compounds, mainly phenolic substances [18].

It is also important to highlight that the sugary fraction derived from hydrolysis of oleuropein during the washing process is susceptible to be transformed into valuable platform compounds, principally 5-hydroxymethylfurfural (HMF) and levulinic acid (LA) [19]. These chemicals possess the capability of generating multiple and interesting products on an industrial level, such as biofuels, solvents, monomers, or pesticides [20,21].

Therefore, the main objective of this work is to show how the chemical valorization of table olive processing wastewater (TOPWW) can generate high-added-value compounds from this type of agri-food waste. The proposed valorization process can be divided into two pathways: the extraction of phenolic compounds and the transformation of the sugary fraction into platform compounds. For this purpose, quantitative NMR (qNMR) has been proposed as a quantification method for both pathways. NMR has increased in popularity as an analytical tool due to how it can provide both qualitative and quantitative data from complex matrices, without tedious sample preparation and in a relatively short time. Thus, NMR is currently employed in other fields apart from the structural elucidation of organic compounds, such as metabolomics in biomedicine [22,23,24,25] and quality control [26,27].

Regarding the application of NMR to the olive industry, both ^1^H-NMR [28] and ^13^C-NMR [29] have been employed for the quantification of several compounds present in olive oils, and especially for minor compounds, such as phenols, sterols, monoacylglycerols, and diacylglycerols, among others. Photis Dais and co-workers have widely studied the application of ^31^P-NMR for the analysis of several analytes, especially phenolic compounds in foods [30,31]. Thus, they developed a methodology to obtain reliable data from the ^31^P-NMR quantification of compounds with labile hydrogens (-OH, -COOH) [32]. It should be noted that, prior to ^31^P-NMR analysis, a derivatization reaction with a phosphorus reagent should be carried out. The reagent employed must react quantitatively with the labile hydrogens of the compound, with 2-chloro-4,4,5,5-tetramethyl-1,3,2-dioxaphospholane (TMDP) being the phosphorus reagent most suitable for the derivatization reaction. The main advantages of this reagent are its lack of formation of isomeric products or side reactions and the stability of the derivatized compounds formed, which remain stable for more than two weeks [33]. Taking this all into account, qNMR represents an interesting option for the determination of the phenolic compound concentration in TOPWW samples.

## 2. Materials and Methods

### 2.1. Chemicals

Sodium hydroxide, sodium chloride, aluminum chloride hexahydrate, *p*-toluensulfonic acid monohydrate (≥98.5), 2-chloro-4,4,5,5-tetramethyl-1,3,2-dioxaphospholane (95%), pyridine, chromium(iii)acetylacetonate, oleuropein, tyrosol, levulinic acid, 5-(hydroxymethyl)furfural, 3-(trimethylsilyl)propionic-2,2,3,3-d4 acid sodium salt, and pyrazine were purchased from Sigma-Aldrich (St. Louis, MO, USA). Hydrochloric acid 37% was provided from Panreac (Barcelona, Spain). Ethyl acetate was obtained from Scharlau (Barcelona, Spain) while maleic acid, CDCl3 (+0.03% vol. TMS), deuterium oxide, and cyclohexanol were purchased from Fluka™ (St. Gallen, Switzerland), VWR Chemicals (Solon, OH, USA), and Acros Organics (Geel, Belgium), respectively.

### 2.2. Experimental Design

**Figure 1:**
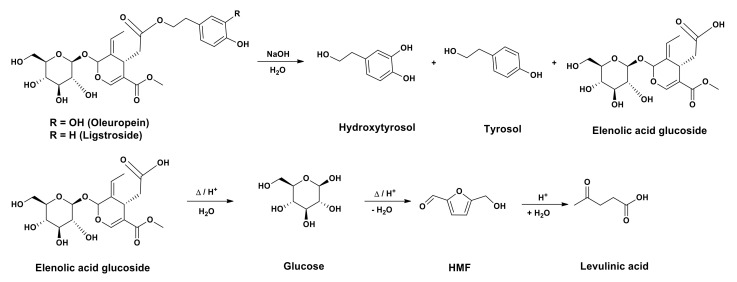
Hydrolysis of oleuropein/ligstroside and further conversion of the elenolic acid glucoside into 5-hydromethylfurfural and levulinic acid.

In this study, the treatment of table olives with aqueous lye (NaOH) solution provoked the cleavage of the oleuropein/ligstroside ester bond, releasing an elenolic acid glucose fragment and a phenol moiety (tyrosol from ligstroside and hydroxytyrosol from oleuropein) [34]. Elenolic acid glucose was also susceptible to hydrolysis, generating glucose, which could be transformed into valuable platform compounds (LA and HMF) [19], as shown in Figure 1.

The initial hydrolysis of oleuropein/ligstroside was performed by immersing the table olives in an aqueous NaOH solution, which breaks the aforementioned bond and extracts the hydrolysis fragments into the aqueous medium. After 24 h, the lye solution that contained the hydrolysis fragments (tyrosol, hydroxytyrosol, and elenolic acid glucoside) was separated from the table olives and noted down as S1. Then, the table olives were immersed in hot, clean water for 12 h. This washing step was aimed at removing the excess NaOH remaining in the olives and continued extracting tyrosol, hydroxytyrosol, and elenolic acid glucoside. The hydrolysis of oleuropein/ligstroside was still active during the washing step because of the action of the residual NaOH remaining in the olives. After 12 h, the washing water was removed and noted down as S2, while the olives were immersed again into clean water. The washings were repeated for the same time (12 h) for up to 2 days, thus obtaining 4 wastewater samples (S2, S3, S4, and S5).

The phenolic compounds tyrosol and hydroxytyrosol were extracted from the TOPWW samples using ethyl acetate (EtOAc). This solvent was selected because both tyrosol and hydroxytyrosol are preferably recovered from olive industry by-products with aprotic and medium polarity solvents. Other compounds with higher polarity, such as oleuropein and elenolic acid glucoside, prefer more polar solvents, such as alcohols and hydro-alcohol mixtures, due to their structure having an elevated number of hydroxyl groups [35]. In recent years, AcEtO has been used for the extraction of tyrosol and hydroxytyrosol from olive industry waste [36,37].

**Figure 2:**
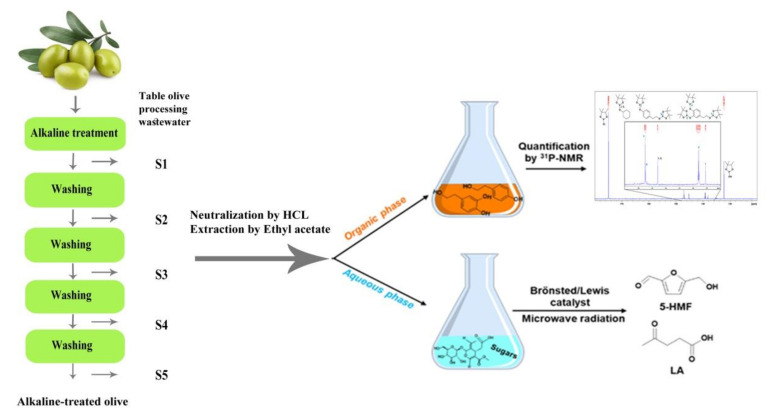
Schematic representation of the valorization of table olive processing wastewater (TOPWW) carried out in this study.

Figure 2 shows a graphical representation of the process. The NaOH treatment hydrolyses the seco-iridoid glycosides oleuropein and ligstroside and the further extraction of the hydrolysis products from the olive pulp, as well as the transformation of the sugary fraction present in the fruit. The compounds present in TOPWW were separated by means of liquid–liquid extraction with EtOAc, obtaining two phases. Furthermore, the organic phase extracted the phenolic compounds tyrosol and hydroxytyrosol, which were further quantified in each TOPWW sample (S1, S2, S3, S4, and S5) by means of ^31^P-NMR (Bruker Ascend™ 400 MHz spectrometer, Bruker Corporation, Billerica, MA, USA). By contrast, the sugary compounds, mainly glucose and elenolic acid glucoside, remained in the aqueous phase. In the case of S1, these sugary compounds were both transformed into platform compounds HMF and LA, using microwave radiation as a heating source (CEM DiscoverSP Monomode microwave reactor, CEM Corporation, New York, NY, USA).

**Figure 3:**
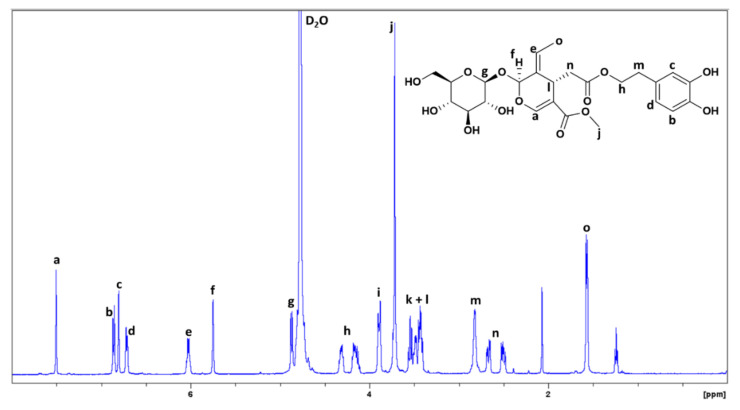
^1^H-NMR spectrum (500 MHz, D_2_O) of oleuropein.

As shown in Figure 3, the 1H-NMR spectrum of oleuropein was useful for tracking the ester hydrolysis of oleuropein/ligstroside and analyzing the NMR spectra of both phases after extraction.

### 2.3. Wastewater Sample Collection and Preparation

The table olives (Gorgal Sevillana variety) used in this study were collected from a local plantation in Guadalmez village within Ciudad Real province (Spain). The samples S1–S5 were obtained by removing the different water solutions + NaOH with which table olives were washed. Treatment was carried out with a dissolution of NaOH 45 g/L of water for 24 h at room temperature (S1), followed by successive washes with water every 12 h (S2–S5).

### 2.4. Sample Extraction

Samples (40 mL) were treated with 12 M HCl until neutralization and extracted with EtOAc (3 × 25 mL) in a 200 mL extraction funnel. A small amount of saturated dissolution of NaCl was added to break down emulsions that may have formed in the funnel. Once separated, both the organic phase (OP) and aqueous phase (AP) reactions were carried out in the rotary evaporator (40 °C) to remove the solvent.

### 2.5. Methodology for Extract Analysis

A fraction of the solid obtained from OP of sample 1 (S1) was taken and analyzed through ^1^H-NMR. Then, the quantification of tyrosol and hydroxytyrosol was performed using quantitative ^31^P-NMR for OP of all the samples (S1–S5). The solid obtained from AP of S1 was analyzed through ^1^H-NMR and quantitative ^1^H-NMR. That solid was also treated with different catalysts (AlCl_3_ or *p*-TSA) and microwave radiation (CEM DiscoverSP Monomode microwave reactor, CEM Corporation, New York, NY, USA) to obtain LA and HMF. A quantitative ^1^H-NMR was also employed to calculate the conversion of sugary fractions (glucose and elenolic acid glucoside) into these platform compounds. A Bruker Ascend™ 500 MHz spectrometer (Bruker Corporation, Billerica, MA, USA) was used to acquire ^1^H-NMR spectra, while the ^31^P-NMR spectra were obtained through a Bruker Ascend™ 400 MHz spectrometer (Bruker Corporation, Billerica, MA, USA).

#### 2.5.1. Sample Preparation for ^1^H-NMR Analysis of Organic Fraction and Aqueous Fraction Residues

A 5 mm diameter resonance tube, micropipettes, and D_2_O were required for sample preparation. First, the sample was re-dissolved in 500 μL of D_2_O. The solution was then passed to the resonance tube and was shaken well, employing a vortex mixer (Heidolph Reax Top vortex, Heidolph Instruments, Schwabach, Germany) to assure homogeneity. The resonance tube was then inserted into the 500 MHz spectrometer.

#### 2.5.2. Sample Preparation for Quantitative ^31^P-NMR

**Figure 4:**
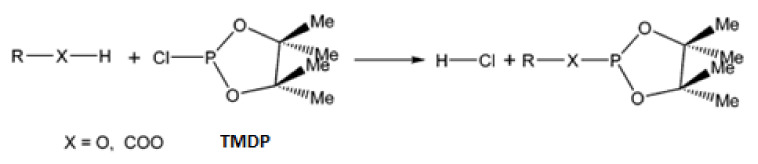
Derivatization process with TMDP.

Initially, a standard dissolution of 25 mL was prepared according to Dais and Spyros [32]. For that purpose, 15.40 mL of pyridine and 9.60 mL of CDCl_3_ (1.6:1 *v*/*v*) were added. Afterwards, 36 μL of cyclohexanol was added as an internal pattern (13.47 mM) and 1.44 mg of chromium acetylacetonate as a relaxing agent (0.165 mM), and, finally, a molecular sieve was included to prevent pyridine from absorbing water. The sample was then prepared in the tube for analysis. Thus, 550 μL of the previously elaborated standard dissolution was added to a flask that contained dried OP. After shaking the flask, the entire volume was passed to the resonance tube, and 60 μL of TMDP was added for the derivatization of the compounds with labile hydrogens present (Figure 4). The tube was then shaken to mix the reagents well and a time of approximately 30 min was expected for the reaction to take place quantitatively. After this time, the tube was inserted into the 400 MHz spectrometer. The experiments were performed in duplicate.

#### 2.5.3. Microwave Single-Phase Reaction

The solid obtained from AP was the one used to conduct the microwave reactions, since it contained the sugary fraction from oleuropein hydrolysis. As a result, 350 mg of sample was transferred to a 10 mL microwave flask equipped with a magnetic stirrer. Then, 2 mL of an aqueous catalyst solution (*p*-toluensulfonic acid or AlCl_3_) of known concentrations, ranging from 0.1 M to 1 M, was added. The flask was sealed and inserted into the microwave reactor, where the reaction was carried out at 190 °C for 20 min. Each reaction was performed in duplicate. After the reaction time, the flask was removed from the microwave and cooled down. It was then filtered into a bend filter, and multiple extractions were performed with EtOAc (3 × 10 mL) to separate the catalyst, which was in the new aqueous phase, from the products, which remained in the organic phase. The solvent was removed from the organic phase in the rotary evaporator, and the solid obtained was subsequently analyzed by ^1^H-NMR.

#### 2.5.4. Sample Preparation for Quantitative ^1^H-NMR after Microwave Reaction

An amount of 500 μL of CDCl_3_ was put in a flask with the reaction product (solid obtained in the previous apart). Then, 50 μL of 0.1 M pyrazine (internal pattern) was added and mixed well before the tube was inserted into the 500 MHz spectrometer.

#### 2.5.5. Acquisition of Spectra

⮚ Proton spectra (^1^H-NMR):

The parameters used in the acquisition of the ^1^H-NMR spectra were relaxation time of 25 s, pulse angle of 90′, acquisition time of 3.277 s, and 8 scans [38]. The signal of TSP (when the dissolvent is D_2_O) was set at 0 ppm, and the signal of TMS contained in the solvent when this is CDCl_3_ was equally set to 0 ppm.

⮚ Phosphorous spectra (^31^P-NMR):

The parameters used for the acquisition of the ^31^P-NMR spectra were relaxation time of 25 s, pulse angle of 90′, acquisition time of 0.63 s, and 160 scans [27].

#### 2.5.6. Data Processing

The obtained NMR spectra were processed using the software TopSpin 3.6.2. (Bruker Corporation, Billerica, MA, USA), and the necessary values were obtained for the realization of the calculations.

#### 2.5.7. Quantification by Quantitative NMR

To perform the quantification, qNMR was used as an absolute method. The quantification of tyrosol and hydroxytyrosol was carried out by ^31^P-NMR, while the quantification of the species formed in microwave reactions was conducted with ^1^H-NMR, as previously mentioned. For quantification, it is necessary to select and measure two integrals: one from a signal associated with the compound of interest and one from a signal related to the internal pattern [38,39]. Then, the following equation is applied (Equation (1)):


(1) $$mx=\frac{Ix}{Ipi}\times \frac{Npi}{Nx}\times PMx\times Nº\;mol\;pi$$

Equation (1) Calculation of the mass of a compound through qNMR [38].

Here, IX and Ipi are the integrals of the compound of interest (x) and the internal pattern (pi), respectively; Nx and Npi are the nuclei for which the selected signals integrate; PMx is the molecular weight of the species under study; Nº mol pi is the number of internal pattern moles added to the experiment; and, finally, mx is the mass of the compound in grams.

## 3. Results and Discussion

### 3.1. ^1^H-RMN of S1 Organic Phase (OP) and S1 Aqueous Phase (AP) after Extraction

**Figure 5:**
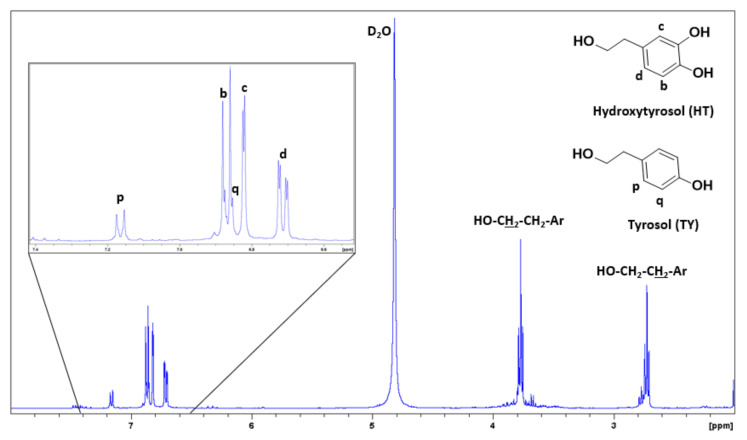
^1^H-NMR spectrum (500 MHz, D_2_O) from S1 organic phase after ethyl acetate extraction of table olive processing wastewater (TOPWW).

Figure 5 shows the ^1^H-NMR spectrum of OP after extraction for S1. It can be clearly seen that the extraction was successfully and that it selectively extracted tyrosol and hydroxytyrosol to a great extent, since no signals from the elenolic acid glucoside fragment or sugars were observed.

**Figure 6:**
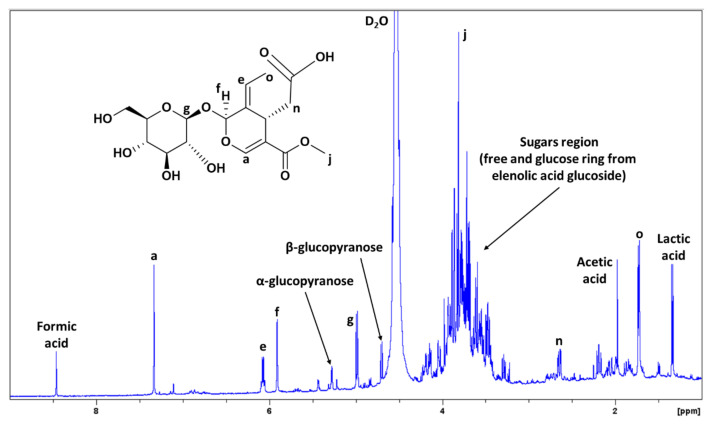
^1^H-NMR spectrum (500 MHz, D_2_O) from S1 aqueous phase after ethyl acetate extraction of table olive processing wastewater (TOPWW).

In addition, Figure 6 shows the ^1^H-NMR spectrum of the S1 aqueous phase after extraction. This time, the main signals observed were those of elenolic acid glucoside, as well as sugars such as glucose. Apart from glucose, xylose was also observed in the spectrum, but to a lesser extent. Finally, other compounds observed were amino acids (small signals between 1–3 ppm), and organic acids such as formic, acetic, and lactic acids. The presence of these organic acids could be related to the decomposition of the sugars present in the table olives.

The aqueous residue of S1 after extraction was also subjected to quantitative ^1^H-NMR to calculate the amount of glucose and elenolic acid glucoside, which are the moieties that can be transformed into 5-HMF and LA. The amount of each compound calculated by means of quantitative ^1^H-NMR was:

⮚ Elenolic acid glucoside: 3.32 wt.%
⮚ Glucose: 1.60 wt.%

It is worth mentioning that the aqueous residue was comprised of NaCl to a great extent, formed during the neutralization step prior to EtOAc extraction, and, therefore, the low percentages of glucose and elenolic acid glucoside were within the expected range. Once the percentage of glucose and elenolic acid glucoside in the sample is known, the amount of glucose existing in a given quantity of sample can be estimated, considering that each mol of elenolic acid glucoside can release a mol of glucose through hydrolysis (Figure 1). Therefore, it is possible to calculate the LA or HMF molar yield as their conversion from glucose is mol to mol.

### 3.2. Quantification of Phenolic Compounds through Quantitative ^31^P-NMR

**Figure 7:**
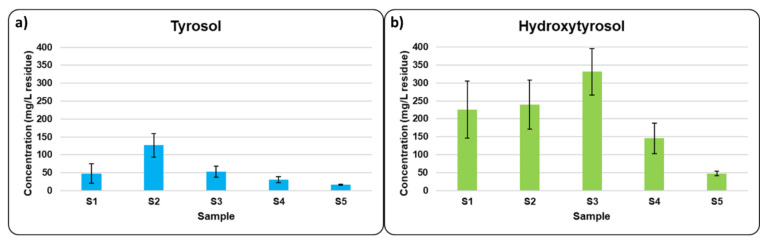
Tyrosol (**a**) and hydroxytyrosol (**b**) content in each table olive processing wastewaters sample.

The results obtained for the quantification of tyrosol and hydroxytyrosol for each TOPWW sample are illustrated in Figure 7. It can be clearly seen that in all samples, the hydroxytyrosol content was between 2 to 6 times higher than the tyrosol content, depending on the sample. This fact is understandable since hydroxytyrosol comes from oleuropein hydrolysis, which is the main seco-iridoid glycoside present in olive tables, whereas tyrosol comes from ligstroside, present in table olives to a lesser extent than oleuropein [24]. These results represent a clear benefit of TOPWW valorization, as hydroxytyrosol is among the phenolic compounds with the highest antioxidant activity [40].

Regarding tyrosol, the first washing wastewater sample (S2) contained higher tyrosol content than the debittering wastewater (S1) (48 mg/L residue vs. 126 mg/L residue), even though S1 was in contact with table olives for 24 h and S2 for 12 h. This trend was also observed by other authors [41] and is due to oleuropein/ligstroside hydrolysis occurring during the debittering stage, whereas hydroxytyrosol/tyrosol were principally removed during the washing step. Moreover, this point also revealed that the hydrolysis of these compounds continued to occur in the table olives during the washing steps. From the second washing step (S3), the tyrosol content decreased and, after four washings (S5), its concentration was 16 mg/L residue.

**Figure 8:**
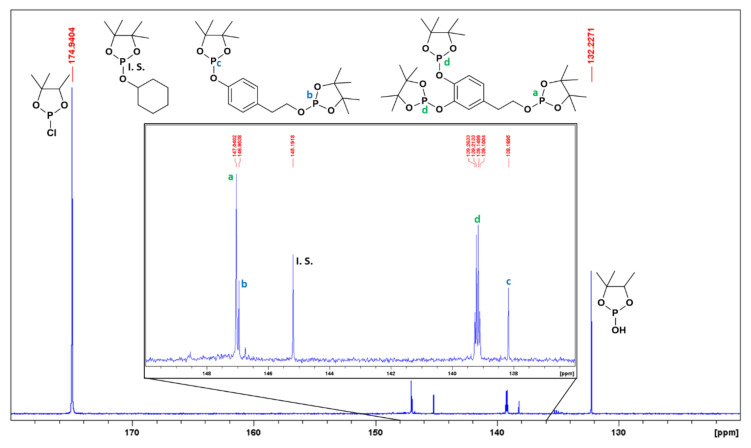
^31^P-NMR spectrum (400 MHz, CDCl_3_ + pyridine) from S1 organic phase after ethyl acetate extraction and derivation process with TMDP.

Concerning hydroxytyrosol, the same trend as previously noted for tyrosol was observed, as the two first washing wastewaters (S2 and S3) contained a higher amount of hydroxytyrosol than the debittering wastewater (S1), with S3 being the sample with the higher concentration of hydroxytyrosol (331 mg/L residue). From S3 on, the hydroxytyrosol concentrations decreased to a value of 48 mg/L residue in S5. Thus, in view of the results obtained for both tyrosol and hydroxytyrosol, it is possible to establish the potential for TPOWW as a source of interesting phenolic compounds. By way of example, Figure 8 shows the ^31^P-NMR spectrum from the analysis of the organic phase of S1.

### 3.3. Conversion of Sugary Fraction from S1 into Platform Compounds

As mentioned above, the resulting aqueous fraction after AcEtO extraction of TOPWW samples contained sugary compounds. The main compounds present in this fraction were glucose and elenolic acid glucoside, resulting from both oleuropein and ligstroside hydrolysis. Elenolic acid glucoside contains a glucose molecule, and, therefore, this residue under acidic conditions could be converted into HMF and LA, according to Figure 1.

The molar yields obtained for the conversion of this sugary fraction into LA and HMF are indicated in Table 1. *p*-TSA and AlCl_3_ were tested to assess which one performed better in the conversion of this residue. Without catalysts, neither HMF nor LA were detected. Reaction without catalysts was performed due to the ^1^H-NMR of the aqueous residue, prior to microwave treatment showing, the presence of lactic and formic acid (as shown in Figure 6). Therefore, we assessed whether these acids could cleave the ether bond in the elenolic acid glucoside and subsequent glucose dehydration into HMF and LA.

For both catalysts, platform compounds were detected when acid catalysts were added. It is worth mentioning that only *p*-TSA 0.1 M afforded HMF, but at low yields (3.66%). For other acid concentrations, the only compound obtained was LA, as was expected, because the aqueous acidic media promote the conversion of HMF into LA [19]. The highest yield of LA was obtained at a 0.5 M acid concentration for both catalysts, although *p*-TSA afforded higher LA yields than AlCl_3_ (69.35% vs. 40.13%, respectively). Finally, high acid concentrations (1 M) provoked a decrease in the LA yield. It is probable that, due to high acid concentrations, carbon-based macromolecular substances such as humins were produced, resulting in losses of the products of interest [42].

Thus, although in some conditions HMF is obtained in a small quantity, the main product observed in this analysis is LA, which is a very interesting valorization product due to the great potential of this platform compound [21].

## 4. Conclusions

In this work, a new strategy for valorizing table olive wastewater was developed, and some valuable components of this waste were identified and quantified by means of NMR spectroscopy. Two main fractions were separated from wastewater samples obtained by NaOH treatment and following washing steps. High amounts of valuable dietary phenolic compounds such as tyrosol and hydroxytyrosol were detected in table olive wastewater. In this regard, it is important to highlight that hydroxytyrosol showed the highest concentration in the analyzed samples, and that this phenolic substance possesses a remarkable antioxidant capacity. Considerable content of phenolic compounds in wastewater obtained from NaOH treatment (S1) was not observed, indicating that water washing at different steps (S2–S5) is fundamental for the recovery of phenolics. Furthermore, it was also revealed that the glucose present in the sugary fraction can be transformed into levulinic acid by a single-phase reaction under microwave irradiation. Considering the great applicability of the compounds derived from levulinic acid, both the phenolic and sugary fractions of TOPWW represent important means for the valorization of wastewater. In view of these findings, it has been proposed that this study should be conducted at an industrial level in future research, as the developed TOPWW valorization has been proven to render interesting results, and quantitative ^31^P-NMR has proven an effective tool for determining phenolic compound concentrations.

## Figures and Tables

**Table 1:** 5-hydroxymethylfurfural and levulinic acid molar yields (%) depending on the acid concentration.

		Molar Yield (%)	
Catalyst	Concentration (mol/L)	Levulinic Acid	HMF
AlCl_3_	0	0	0
	0.1	14.89 ± 8.47	0
	0.25	14.69 ± 8.05	0
	0.5	40.13 ± 10.38	0
	1	20.59 ± 0.83	0
*p*-TSA	0	0	0
	0.1	0	3.66 ± 0.4
	0.25	9.66 ± 4.02	0
	0.5	69.35 ± 2.74	0
	1	48.44 ± 4.68	0

## Data Availability

The data presented in this study are available on request from the corresponding author. The data are not publicly available due to containing information that could compromise the privacy of research participants.

## References

[1] Rincón-Llorente B., la Lama-Calvente D., Fernández-Rodríguez M.J., Borja-Padilla R. (2018). Table olive wastewater: Problem, treatments and future strategy. A review. Front. Microbiol..

[2] Economic Affairs & Promotion Unit. https://www.internationaloliveoil.org/what-we-do/economic-affairs-promotion-unit/.

[3] Gavahian M., Khaneghah A.M., Lorenzo J.M., Munekata P.E.S., Garcia-Mantrana I., Collado M.C., Meléndez-Martínez A.J., Barba F.J. (2019). Health benefits of olive oil and its components: Impacts on gut microbiota antioxidant activities, and prevention of noncommunicable diseases. Trends Food Sci. Technol..

[4] Jimenez-Lopez C., Carpena M., Lourenço-Lopes C., Gallardo-Gomez M., Lorenzo J.M., Barba F.J., Prieto M.A., Simal-Gandara J. (2020). Bioactive compounds and quality of extra virgin olive oil. Foods.

[5] Žugčić T., Abdelkebir R., Alcantara C., Collado M.C., García-Pérez J.V., Meléndez-Martínez A.J., Jambrak A.R., Lorenzo J.M., Barba F.J. (2019). From extraction of valuable compounds to health promoting benefits of olive leaves through bioaccessibility, bioavailability and impact on gut microbiota. Trends Food Sci. Technol..

[6] Cardoso S.M., Falcão S.I., Peres A.M., Domingues M.R.M. (2011). Oleuropein/ligstroside isomers and their derivatives in Portuguese olive mill wastewaters. Food Chem..

[7] Brenes M., Ramírez E., García P., Medina E., de Castro A., Romero C. (2018). New developments in table olive debittering. Proceedings of the Acta Horticulturae.

[8] Roselló-Soto E., Koubaa M., Moubarik A., Lopes R.P., Saraiva J.A., Boussetta N., Grimi N., Barba F.J. (2015). Emerging opportunities for the effective valorization of wastes and by-products generated during olive oil production process: Non-conventional methods for the recovery of high-added value compounds. Trends Food Sci. Technol..

[9] Putnik P., Barba F.J., Španić I., Zorić Z., Dragović-Uzelac V., Kovačević D.B. (2017). Green extraction approach for the recovery of polyphenols from Croatian olive leaves (*Olea europea*). Food Bioprod. Process..

[10] Şahin S., Elhussein E., Bilgin M., Lorenzo J.M., Barba F.J., Roohinejad S. (2018). Effect of drying method on oleuropein, total phenolic content, flavonoid content, and antioxidant activity of olive (*Olea europaea*) leaf. J. Food Process. Preserv..

[11] Žuntar I., Putnik P., Kovačević D.B., Nutrizio M., Šupljika F., Poljanec A., Dubrović I., Barba F.J., Jambrak A.R. (2019). Phenolic and antioxidant analysis of olive leaves extracts (*Olea europaea* L.) obtained by high voltage electrical discharges (HVED). Foods.

[12] Hadidi M., Amoli P.I., Jelyani A.Z., Hasiri Z., Rouhafza A., Ibarz A., Khaksar F.B. (2020). Polysaccharides from pine-apple core as a canning by-product: Extraction optimization, chemical structure, antioxidant and functional properties. Int. J. Biol. Macromol..

[13] Hesami G., Darvishi S., Zarei M., Hadidi M. (2021). Fabrication of chitosan nanoparticles incorporated with Pistacia atlantica subsp. kurdica hulls’ essential oil as a potential antifungal preservative against strawberry grey mould. Int. J. Food Sci. Technol..

[14] Alcántara C., Žugčić T., Abdelkebir R., García-Pérez J.V., Jambrak A.R., Lorenzo J.M., Collado M.C., Granato D., Barba F.J. (2020). Effects of ultrasound-assisted extraction and solvent on the phenolic profile, bacterial growth, and anti-inflammatory/antioxidant activities of mediterranean olive and fig leaves extracts. Molecules.

[15] Hadidi M., Ibarz A., Pouramin S. (2021). Optimization of extraction and deamidation of edible protein from evening primrose (*Oenothera biennis* L.) oil processing by-products and its effect on structural and techno-functional properties. Food Chem..

[16] Cárdeno A., Sánchez-Hidalgo M., Rosillo M.A., De La Lastra C.A. (2013). Oleuropein, a secoiridoid derived from olive tree, inhibits the proliferation of human colorectal cancer cell through downregulation of HIF-1α. Nutr. Cancer.

[17] Giacomazza D., D’Andrea D., Provenzano A., Picone P., Provenzano F., Guarrasi V., Raimondo M., Biagio P.L.S., Passantino R., Mangione M.R. (2018). The precious content of the olive mill wastewater: The protective effect of the antioxidant fraction in cell cultures. CYTA-J. Food.

[18] Garrido A., Brenes M., García P. (1999). Treatment for green table olive fermentation brines. Food Preserv. Technol. Ser..

[19] Mika L.T., Cséfalvay E., Németh Á. (2018). Catalytic conversion of carbohydrates to initial platform chemicals: Chemistry and sustainability. Chem. Rev..

[20] Wang Y., Brown C.A., Chen R. (2018). Industrial production, application, microbial biosynthesis and degradation of furanic compound, hydroxymethylfurfural (HMF). AIMS Microbiol..

[21] Pileidis F.D., Titirici M.-M. (2016). Levulinic acid biorefineries: New challenges for efficient utilization of biomass. ChemSusChem.

[22] Durán-Prado M., Frontiñán J., Santiago-Mora R., Peinado J.R., Parrado-Fernández C., Gómez-Almagro M.V., Moreno M., López-Domínguez J.A., Villalba J.M., Alcaín F.J. (2014). Coenzyme Q10 protects human endothelial cells from β-Amyloid uptake and oxidative stress-induced injury. PLoS ONE.

[23] Villar M., Ayllón N., Alberdi P., Moreno A., Moreno M., Tobes R., Mateos-Hernández L., Weisheit S., Bell-Sakyi L., La Fuente J. (2015). Integrated metabolomics, transcriptomics and proteomics identifies metabolic pathways affected by Anaplasma phagocytophilum infection in tick cells. Mol. Cell. Proteom..

[24] Frontiñán-Rubio J., Santiago-Mora R.M., Nieva-Velasco C.M., Ferrín G., Martínez-González A., Gómez M.V., Moreno M., Ariza J., Lozano E., Arjona-Gutiérrez J. (2018). Regulation of the oxidative balance with coenzyme Q10 sensitizes human glioblastoma cells to radiation and temozolomide. Radiother. Oncol..

[25] Lucas-Torres C., Roumes H., Bouchaud V., Bouzier-Sore A.-K., Wong A. (2021). Metabolic NMR mapping with microgram tissue biopsy. NMR Biomed..

[26] Casas A., Ramos M.J., Pérez A., Simón A., Lucas-Torres C., Moreno A. (2012). Rapid quantitative determination by 13C NMR of the composition of acetylglycerol mixtures as byproduct in biodiesel synthesis. Fuel.

[27] Lucas-Torres C., Pérez Á., Cabañas B., Moreno A. (2014). Study by ^31^P NMR spectroscopy of the triacylglycerol degradation processes in olive oil with different heat-transfer mechanisms. Food Chem..

[28] Christophoridou S., Dais P. (2009). Detection and quantification of phenolic compounds in olive oil by high resolution ^1^H nuclear magnetic resonance spectroscopy. Anal. Chim. Acta.

[29] Vlahov G. (1999). Application of NMR to the study of olive oils. Prog. Nucl. Magn. Reson. Spectrosc..

[30] Christophoridou S., Dais P. (2006). Novel approach to the detection and quantification of phenolic compounds in olive oil based on ^31^P nuclear magnetic resonance spectroscopy. J. Agric. Food Chem..

[31] Agiomyrgianaki A., Dais P. (2012). Simultaneous determination of phenolic compounds and triterpenic acids in oregano growing wild in Greece by ^31^P NMR spectroscopy. Magn. Reson. Chem..

[32] Dais P., Spyros A. (2007). ^31^P NMR spectroscopy in the quality control and authentication of extra-virgin olive oil: A review of recent progress. Magn. Reson. Chem..

[33] Christophoridou S., Spyros A., Dais P. (2001). ^31^P nuclear magnetic resonance spectroscopy of polyphenol-containing olive oil model compounds. Phosphorus Sulfur Silicon Relat. Elem..

[34] Cavaca L.A.S., Rodrigues C.A.B., Simeonov S.P., Gomes R.F.A., Coelho J.A.S., Romanelli G.P., Sathicq A.G., Martínez J.J., Afonso C.A.M. (2018). Valorization of oleuropein via tunable acid-promoted methanolysis. ChemSusChem.

[35] Galanakis C.M., Goulas V., Tsakona S., Manganaris G.A., Gekas V. (2013). A knowledge base for the recovery of natural phenols with different solvents. Int. J. Food Prop..

[36] De Leonardis A., Macciola V., Lembo G., Aretini A., Nag A. (2007). Studies on oxidative stabilisation of lard by natural antioxidants recovered from olive-oil mill wastewater. Food Chem..

[37] De Marco E., Savarese M., Paduano A., Sacchi R. (2007). Characterization and fractionation of phenolic compounds extracted from olive oil mill wastewaters. Food Chem..

[38] Holzgrabe U. (2010). Quantitative NMR spectroscopy in pharmaceutical applications. Prog. Nucl. Magn. Reson. Spectrosc..

[39] Lucas-Torres C., Lorente A., Cabañas B., Moreno A. (2016). Microwave heating for the catalytic conversion of melon rind waste into biofuel precursors. J. Clean.

[40] Martínez L., Ros G., Nieto G. (2018). Hydroxytyrosol: Health benefits and use as functional ingredient in meat. Medicines.

[41] Parinos C.S., Stalikas C.D., Giannopoulos T.S., Pilidis G.A. (2007). Chemical and physicochemical profile of wastewaters produced from the different stages of Spanish-style green olives processing. J. Hazard. Mater..

[42] Lopes E.S., Leal Silva J.F., Rivera E.C., Gomes A.P., Lopes M.S., Maciel Filho R., Tovar L.P. (2020). Challenges to levulinic acid and humins valuation in the sugarcane bagasse biorefinery concept. Bioenergy Res..

